# Errors in Drug Proving

**Published:** 1887-10

**Authors:** P. P. Wells

**Affiliations:** Brooklyn, N. Y.; Bureau of Materia Medica, I. H. A.


### ERRORS IN DRUG PROVING.

P. P. Wells, M. D., Brooklyn, N. Y.

(Bureau of Materia Medica, I. H. A.)

Success in the practice of specific medicine (Homœopathy) depends so largely on the purity of our *Materia Medica* record that we may safely say, without this there can be no certainty in the results of the best endeavors of the best prescribers. This purity must be in the foundation, in the proving of those agencies upon the healthy life, the record of which contains our science of materia medica. This must be composed of *facts* only, with the conditions, circumstances, and concomitants which attended these when they were observed and recorded. Circumstance, condition, and concomitants are *parts of the facts* of the record which, being omitted, the record is useless for all clinical purposes. They are the indices which point to the *true character* of the facts of the record, and are the chief authority in the decision of the selection of the specific curative for a case of sickness; because in these are found similarity of condition, circumstance, and concomitants related to its elementary symptoms, as these appeared in the history of the case. It is in these that the relationship of law between sicknesses and their specific curatives exists. It is the omission to observe and record these which constitutes the one great and fundamental error of most modern provings, by which they are made to stand as little better than so much dead matter before all clinical needs.

The second great error in our modern provings is in the wrong views provers have entertained of the true objective of the proving. They have regarded this as the results of violent revolutionary processes set up in the functions of the organism, and that the more violent these were in the experience of the prover, the more valuable the proving. Hence, to secure the desired violence of the symptoms, resort has been had to inordinate doses of the drug, which have been but too speedily expelled from the organism, and this before there had been time for the development of the true objective of the proving, viz.: to obtain a knowledge of the true specific character of the action of the drug on the life force of the prover. This gained, we have an exact knowledge of that in the drug which cures. When the drug is expelled by the violence of its own action this knowledge is never attained. It is the knowledge of the specific nature of this action which gives us the characteristics of the drug which are those elements exclusively of use in clinical duties. This knowledge, therefore, is the true objective of drug proving, and not a knowledge of those violent commotions of function which have deprived us of this.

The third error, which has too much characterized modern attempts at provings, is found in the views of the provers of the nature of the essential element in the drug which constitutes it a medicine, and which it is the business of the prover to investigate. These views were limited to the material substance of the drug—to this as matter—and their judgment, on which they proceeded in their task, has been—the more of this matter given, the more numerous and the more important the resulting symptoms will be. This judgment being the exact opposite of the truth, their labors have been, of course, almost wholly destitute of practical value. This view of the proper object of the proving ignores the all-important truth which Hahnemann first discovered and gave to the knowledge of the scientific world—that it is *not* the *matter* of the drug which cures or makes sick, but a dynamic element in it which it pleased the Creator to associate with the material element. That this dynamis, even in the matter of the most virulent poisons, may, by proper manipulation, be tamed and converted into gentle, harmless, and most beneficent healing agents, so that when ingested it no longer destroys life, or sets up in the organism violent commotions for the rapid expulsion of the destructive agent. So manipulated, it is tolerated by the organism, and its specific impression on the vital dynamis reveals its own *specific* character to the prover and gives for the use of the true healer the knowledge which enables him to triumph over sickness, under the guidance of the law of similars. This knowledge, so obtained, makes a therapeutics founded on it, and administered according to law, triumphant over all curable sicknesses. While, on the other hand, when regarded only as a material agent, the prover gains no light on this specific nature of the action of the drug, because the process of the proving is ended by the violence of the crude and massive doses employed before these specific effects are reached.

In view of these truths, the ignorance and folly of those would-be reformers of our materia medica who reject from its record all symptoms not the product of low dynamizations or of crude doses of the drugs proven, is perfectly apparent. By this scarcely less than idiotic proceeding they exclude from the record elements of the greatest value, and leave only those which, in comparison, in therapeutic value are little better than dead husks. So great damage is wrought when audacious folly substitutes its own attempts at a philosophy of therapeutics for that pertaining to the therapeutics of the law of similars. It strips the record, from which those who have been our pioneers of healers have drawn the knowledge which has enabled them to achieve their great successes, of its chiefest wealth. And yet these wrong-doers would have the world regard them as authoritative representatives of that which in their labors they have been only active in endeavors to destroy. They affected a reform, and so far as their endeavors have had efficiency they have only wrought a destruction. It could not be otherwise when those who start to investigate nature begin by themselves resolving what shall and what shall not be found in her! Perhaps human silliness has gone no further in any associated combination than when our "great representative body" took upon itself to say where curative power, in medicinal preparations, should and should not be found. This so ridiculous folly was possible only as the outgrowth of the error in philosophy which substitutes the matter of the drug for its dynamis, which alone relates it to human sickness.

The fourth error in provings has been in the *dose* and its *management*, and is a legitimate outcome of errors two and three, which we have already considered. If that to be questioned be the matter of the drug, what can be more reasonable than this, there must be enough of it to make itself *felt*, and to make it felt so immediately as to give certainty that that which *is* felt is

*caused by the drug taken?* And then, the more of that which is felt, the more numerous and the more valuable its result in symptoms to be added to our materia medica record. This has been the course of thought of so many who would march over the heads of the great, wise, and successful masters who preceded them, and pose as reformers and "*re-provers*" of our materia medica. And in acting on this thought they have given and taken doses which have made sick sure, and they have made sick soon, and there was no chance even for skepticism to doubt the sickness was caused by the dose. But then, *cui bono*, who is the better for all this? There is in this violent and brief commotion little or nothing of value to therapeutics. The elements of the sicknesses thus produced are only representatives of the poisonings of drugs, and therefore belong to the *destructive* side of its nature, and but little or not at all to that of its healing power. So by all this false philosophy and wrong practice based upon it, we have only a record of sufferings which profit no man or interest. And then, under the impulse of this false philosophy, the evil is exaggerated by the management of the doses employed. "Give enough," is the order, and *repeat* often enough to keep up the storm raised, and long enough to exhaust all there is in the experience of the action of the drug, and then what have we but a proving? This process insures anything else, and its results have proved this so many times and so often, that the world, and especially those in it who will engage in the work of drug proving, should long ago have been cured of the foolishness of their false philosophy, and of their blindness, which has tortured self and friends with the best of motives, but with so little profit to any.

If there is to be a proving of any drug which is to add to any man's knowledge of the characteristics of the agents he is to employ in clinical duties, it must be introduced into the organism in manner and form that will insure the tolerance of its presence without violent commotion of functions which follow the ingestion of massive, crude, and frequent doses. It must be in the form which will permit the absorption of its dynamis into that of the force which executes and governs the bodily functions, that by its action on this force it may so modify these functions as to declare in these modifications, its own specific character. In the record of these modifications, and in these only, we have a *true proving* of the drug, a proving which is available for all time for the relief of the sick and suffering of mankind.

When the potentiality of the drug has been thus introduced to the life force, it is to be let alone that its true character may be fully revealed. It is not to be disturbed by any other dynamis, nor by any repetition of doses of itself, until there has been sufficient time for the original dose to exhaust its action. This, not infrequently, embraces days, weeks, and even months. Care in this is the more important because it has been learned by the ablest students of materia medica (Bœnninghausen, Hering) and the best observers of drug proving, that the symptoms of the greatest value in a proving are those *last* developed. By any too early interference with the progress of the action of the drug its most important symptoms are lost to the record. This time, as we have said, sometimes extends to months. Then, asks the prover with crude drugs and doses, who only sees or can see that which immediately follows ingested doses of his drugs, " How are we to know that those far-off facts *are* the effects of the claimed dynamis, given so long ago ?" It is quite natural and reasonable that he should ask this, because he does not know. He has never seen such symptoms, *his methods* of proving have precluded this, and his material philosophy of drug nature and action has deprived him of the only light which could have shown him the connection of these long-deferred symptoms with the cause which has produced them.

In order to see this connection plainly it may be necessary to go back to the fundamental element of drug philosophy, the universality of the dynamic element which pervades all forms of matter; to bear in mind that each form of matter has a dynamic element all its own, which possesses specific characteristics of nature, which give it an individuality which distinguishes it from the dynamis of all other forms of matter; that in the proving of this drug it impresses this individuality on the modified actions of functions through its specific action on the life force which executes them, and this to the degree that experts see this specific quality running through the series of developed facts, which they have likened to the golden thread which has been twisted into the many-stranded cord. This specific quality found in the condition, circumstance, or concomitants of any experienced symptom, and probability is so stamped on that symptom as a legitimate child of that drug. If on repetition of the experiment there results a repetition of the symptom with its former characteristics, probability is converted into a confirmation, and then, if on this basis the drug be given to one sick who is showing similar phenomena in his symptoms and the sick one be cured by the dose, confirmation becomes assurance, and the long-delayed and questioned symptom may be accepted into our materia medica without hesitation. Or, if the questioned symptom be but slightly marked by the specific character of the drug, or even if this be not recognized at all, and yet the symptom is repeated in the experience of different provers, and its genuineness be proven by the successful use of the drug in clinical cases characterized by similar symptoms, then the symptom may be safely accepted as a legitimate element of a pure materia medica. The factor of time between the ingestion of the drug and the advent of the symptom, however protracted, is not to exclude the fact nor diminish our confidence in it as an index to a needed curative.

Then there may be errors in making up the record of the experiences of provers. The value of their labors is wholly in this element of purity. It is to be made up of *all* the facts which have had their origin in the action of the drug on the life force of the prover, and of no others. No facts of this class are to be omitted because the prover, or his recorder, judges them to be of little importance. It is to be a law controlling this record that no *fact* is unimportant because it has been attended with little suffering or danger to the prover. It may be of little significance to the diagnosis, while it is of the greatest value to therapeutics. What can be apparently more trivial than the sensation "tickling on the face *as if from a hair*"? And yet this was recorded, and afterward became the deciding factor in the selection of this remedy by one of our greatest masters in one of his greatest cures. Or its near of kin, "sensation as of a hair on the tongue,"* which was ridiculed as an example of the supremely silly in a session of the American Institute by a member who had been before, was then, and has been ever since ambitious of the functions of a materia medica *reformer!*

A less ambitious, but more intelligent, member (Dunham) saw in this ridiculed fact an exceedingly important warning of the approach of a very grave disease.

This would-be reformer, who could see no importance in this fact—indeed, he could not see that it was a fact—has since tried his hand at his coveted work, and the result has proven a greater silliness was possible than his failure to comprehend the significance and import of a single drug symptom. The weakness and silliness of his work is only surpassed by the sadness

* Sensation of tickling on back part of tongue, not relieved by eating or drinking, *Kali-b.;* on fore part is Silicea. Tickling on face as from a hair, Laur. —EDS. H. P.

compelled by the damage wrought by ignorance and folly on the noblest labors of the noblest of men.

When we say purity of the record of a proving requires that *all* the experienced facts of that proving shall be carefully and truthfully written therein, we mean all symptoms of both body and *mind*.

We note this more particularly because it is one of the gravest errors of modern provers that these most important symptoms to the therapeutist are either omitted altogether, as a matter of no concern to the prover, or disposed of so slightingly and carelessly as to add little of value to a record this omission or carelessness has so nearly made valueless. It is these mental symptoms, so slighted or neglected by the ignorant or unskillful prover, which often are the elements in a pure record which decide the differentiation of similar remedies in the process of prescribing. For example, who with even the least intelligence of applied materia medica, would think of giving Pulsatilla to a proud and haughty patient, or Aconite to a tranquil and submissive sufferer? The prover should never forget in his proving and in its record, if any class of symptoms is more important to the true objective of the proving than another, it is that of the mental and moral sphere. These omitted or marred, and the proving has no place in a *pure* materia medica record.

Purity of materia medica record means not only no omitted facts, which have been developed in the experience of the prover while under the action of the drug he is proving, which can legitimately be put to the account of the drug for any reason whatever, but also no fact is to be entered on the record which has had its origin in any other cause. Impurity then is found equally in the *absence of facts* as in the intrusion of the false. If facts arise in the progress of the proving of which there is doubt as to their cause, whether they be of the drug, they are not, therefore, to be rejected, and, of course, they are not to be accepted, till the doubt is solved by further experiment and observation. The doubt is dissipated most perfectly by the clinical test. Does the drug cure or fail to cure similar symptoms in the sick? If it cure, the symptoms are confirmed as of a right belonging to the record, beyond the possibility of a doubt. But till confirmed by the clinical test or by further experiment and observation, they can only have place with the facts recorded as still under observation.

We do not forget that the clinical test of the action of drugs has been rejected as authority in the affirmative of this question. Nor do we forget that unreasonable and unreasoning skepticism has been driven by unquestionable facts to this near approach to idiocy by its persistent refusal to accept demonstrated facts which admitted of no other negation. The resort to this is perfectly *safe* to those who flee to it, as by this they refuse to come before the *only* tribunal where the questioned truth can be tried and decided. It leaves them to their *non credo*, and beyond this they will never go, so great is the folly of *skepticism of the will!* Incurably weak and blind!

A *pure* materia medica is the only objective of drug proving. By *pure* in this connection we mean a record of *all* the facts which the drug has been found to produce when brought into contact with the life force of man. The record, to be PURE, *i. e.*, perfect, is to be free of all facts not the effects of the drug, whatever the origin of these may be, and to secure this freedom there needs to be on the part of the prover and recorder especial care to avoid these oscillations of function which are constantly occurring by reason of disturbances of functional balance from other natural causes than the drug. These are met more or less in every individual who is not in *perfect* health, and those who are gifted with this chiefest of earthly blessings are not numerous. Where these spontaneous disturbances are frequent and many they disqualify altogether for the work of the prover.

There is necessary to this desired perfect record not only absence of all which is *false*, but also the presence of *all* which is *true*. If there be many symptoms in the record of any drug, this abundance is no warrant for the exclusion of any—though there have been those who have proposed, as a reform of our materia medica, that the record of "no drug should be allowed to exceed *five pages*." Mangling, mutilating, and reform were evidently synomyns in the mind of this chairman of the bureau of the "great representative body"! We have already seen that circumstance, condition, and concomitants of each symptom were necessary to its perfect record. But this is not all. Quality of symptoms, as of pains, of which kind are they? where is the exact location of each? what is its character? is it fixed or moving? if moving, does it shoot from point to point? if so, in what direction are its dartings? are they from above downward, or the reverse? or are they transverse? if so, from which side do they start? or do they shift from one location to another? if so, was the transit from right to left, or the reverse? or was it from below upward, or the reverse? These and all else which has characterized the advent, presence, or disappearance of any symptom is to be made a part of the record which is pure, *i. e.*, perfect.

And, finally, purity of the record requires of the prover that he enter upon his work with no preconceived notions of what is or is not to be. He is only to concern himself with that which actually happens, with that which *is*. He has no authority to say of this or that that it could not have its origin in the dose because it was of this or that dynamization. He has neither knowledge nor power which can determine the limits of drug action on the life dynamis. This knowledge and power Omniscience and Omnipotence has reserved to itself, and, so far as we know, has shared it with no materia medica reformers. And, moreover, the prover is not to be frightened by any, no matter how great, "wilderness of symptoms" he may develop. He is only to be concerned with one and all, with few or many, from crude drugs or dynamizations, high or low, with the answer to this one question—is it true? Is it a representative, *quo ad hoc*, of the action of the drug under examination?

